# Beneath the calm waters: unveiling the unseen emotional labor in the outsourced interpreting

**DOI:** 10.3389/fpsyg.2026.1728184

**Published:** 2026-07-08

**Authors:** Zhourong Shen, Bin Gao

**Affiliations:** 1 School of Interpreting and Translation, Beijing International Studies University, Beijing, China; 2 School of English and International Studies, Beijing Foreign Studies University, Beijing, China

**Keywords:** emotional dissonance, emotional labor, Hangzhou Asian Games, interpreter, outsourced interpreting

## Abstract

**Background:**

Emotional labor in interpreting has been examined primarily within the boundaries of interpreted encounters, leaving the periods surrounding such encounters underexplored. This gap is particularly significant in outsourced interpreting, where interpreters operate under a dual authority structure. Interpreters have to deal with intensified occupational stress and emotional dissonance as they are simultaneously accountable to language companies and commissioning institutions. How interpreters manage their emotional responses across the full temporal trajectory of an outsourced contract remains insufficiently understood.

**Methods:**

This study examines the emotional labor of 52 professional Chinese-English interpreters during the 19th Hangzhou Asian Games in 2023 using a sequential explanatory mixed-methods design. The author, embedded in the cohort as both researcher and interpreter, adopted an autoethnographic approach. Sentiment analysis was conducted over group chats along with frequency and TF-IDF analyses to identify emotion-signifying markers. Semi-structured interviews were conducted with two purposively selected interpreters representing contrasting emotional trajectories, with findings used to contextualize and interrogate the sentiment patterns.

**Results:**

Sentiment scores were lowest during the Games period (2.48), consistent with heightened emotional dissonance under dual institutional supervision. The use of mitigating expressions peaked during this period, suggesting widespread surface acting in interpreter-language company interactions. Sentiment scores peaked on the final day of the Games (7.90), reflecting behavioral patterns consistent with deep acting and professional inculcation. Interview data revealed that interpreters experiencing emotional dissonance during interpreting subsequently disclosed genuine emotions to trusted peers in inter-interpreting settings as a coping and resource-replenishment strategy, while carefully managing their self-presentation toward the language company throughout.

**Conclusion:**

Outsourced interpreting imposes compounded emotional labor demands extending well beyond interpreted encounters. This study introduces the distinction between intra-interpreting and inter-interpreting settings as a conceptual framework for mapping this temporal ecology, and demonstrates that interpreters engage in both visible and invisible forms of emotional labor to mitigate the negative effects of power asymmetry on their well-being and professional practice.

## 1 Introduction

There is a growing trend for institutions to outsource interpreting services to private companies rather than hiring qualified professionals directly (Vigier-Moreno, 2020, pp. 208). Consequently, interpreters increasingly contract with language companies. This shift has been noted in the literature. Highlighting that institutions are on a race to cut cost and improve productivity, Moorkens (2017, p. 2) argues that translation industry is moving towards contract work and task-based payments, which compromise job security, rights, and agency for workers. For freelance interpreters frequently obtaining assignments from language companies, outsourced interpreting has become their everyday reality, altering their mindsets in work relationship management.

Concerns have been raised about outsourced interpreting, which involves new types of collaboration among partners and agents with varying aims, expectations, standards, and working methods (Drugan, 2020, pp. 308). In this context, it is unsurprising that interpreters feel less confident in their status, as they are managed by agencies rather than serving as direct service providers. In addition, interpreters are finding their fees drastically decreased as language companies are maximizing profits at their expense, despite being essential allies in providing professional services (García-Beyaert, 2015, pp. 54). Interpreters seem to have very little power to revert the situation (Vigier-Moreno, 2020, pp. 222).

Outsourcing services could prove to be beneficial to institutional well-being, but it may also lead to several problems, including losing control over the outsourced activity and overlooking of personnel issues (Baitheiemy, 2003, pp. 87). The well-being of interpreters in outsourced interpreting is often overlooked. Interpreters not only have to perform tasks involving power differentials with speakers (Angelelli, 2004; Morris, 2010; Becher and Wieling, 2015), but also adhere to the directives of the commissioning institutions (Rudvin, 2005). In institutional terms, interpreters enjoy only very limited power (Mason and Ren, 2012, pp. 238). Now interpreters also have to answer to language companies that take commissions from their remunerations. Zwischenberger and Alfer (2022, pp. 218) note that professional translators are more aware of the economically exploitative structures of the translation market, but can off-set contractual disenfranchisement with personal satisfaction and a deep sense of the meaningfulness of their work. However, it remains unclear how interpreters manage the negative effects of this two-fold power asymmetry beyond interpreting tasks.

The fulfilment of interpreting task intrinsically involves emotional labor (Ayan, 2021, pp. 125). In outsourced interpreting, institutions and language companies prescribe work norms control interpreters’ remuneration, work schedules, and logistics. Expressing organizationally desired emotions that are not genuinely felt creates emotional dissonance, leading to greater emotional exhaustion and lower job satisfaction (Morris and Feldman, 1996, pp. 986). Interpreters often remain silent about stringent work requirements and unsatisfactory arrangements, further contributing to negative emotions. Managing the emotional dissonance induced during interpreting and coping with undesirable work conditions require additional emotional labor for reconciliation. This effort, however, often remains unseen as it only manifests after interpreting events.

This article examines the emotional labor of interpreters at the 19th Hangzhou Asian Games in 2023, an event involving 52 professional Chinese-English interpreters. The Organizing Committee outsourced language services to a language company through a bidding process. The Committee sets up stringent quality standards on interpreters and communicates its evaluations through the language company, which also assess interpreters through a quality control manager. This dual supervision mechanism creates significant institutional pressures for interpreters. Challenges during interpreting, negative evaluations and unsatisfactory work arrangements often lead to emotional dissonance. The emotional effort processing this dissonance often goes unnoticed, as it occurs in less conspicuous settings and manifests subtly. Unlike conventional conferences where interpreters are confined to booths scattered across different venues, the Asian Games established a remote interpreting center with a dedicated open-plan office. This setup made interpreters’ behaviors and interactions observable, providing unique insights into the emotional complexities of their professional practice.

This study is guided by three research questions. First, how do interpreters experience emotional labor in outsourced interpreting? Second, how does outsourcing influence interpreters’ emotional labor? Third, what forms of emotional labor occur in inter-interpreting settings beyond the immediate interpreting encounter? Addressing these questions, this article aims to unveil the unseen dimensions of interpreters’ emotional life in an ever-increasingly outsourced field.

## 2 Understanding interpreter’s emotional labor

### 2.1 Emotional labor as a theoretical underpinning

Emotional labor refers to the management of feelings to produce publicly observable facial and bodily displays, requiring individuals to induce or suppress feeling in order to sustain a proper outward countenance (Hochschild, 1983; Hochschild, 2012, pp. 7). Occupations involving emotional labor typically share three characteristics: face-to-face or voice-to-voice interaction with the public, the production of emotional states in others, and employer control over emotional conduct through training and supervision (ibid Liu, 2012, pp. 147). Recognizing that emotional labor is an observable behavior rather than just an internal state, Ashforth and Humphrey (1993, pp. 89) distinguish surface acting, deep acting, and the expression of genuine emotion as three means of feeling management. Surface acting, which involves feigning emotional expressions, has been consistently associated with emotional exhaustion, job dissatisfaction, and psychological distress (Grandey, 2000, pp. 97). Deep acting, defined as modifying one’s actual feelings through self-induction (Grandey and Gabriel, 2015), is more closely associated with job satisfaction and personal accomplishment, yet may still contribute to withdrawal and negative work attitudes (Yang and Chen, 2021, pp. 484). By contrast, the expression of genuine emotions can reduce exhaustion and promote emotional consonance when aligned with institutional display rules (Mesmer-Magnus et al., 2012, pp. 8).

Emotional labor constitutes an integrated process involving emotional job demands (environmental stimuli), emotion regulation (intrapsychic responses), and emotional performance (interpersonal behavior) (Grandey and Gabriel, 2015, pp. 21.5). This framework is particularly relevant to interpreting, where affect, cognition, and behavior are intrinsic to social interaction (Shih and Wang, 2024). Interpreting is psychologically demanding, requiring interpreters to deploy emotional responses to manage stress during performance (Korpal and Jasielska, 2019, pp. 4). Bontempo and Napier (2011) identify emotional stability as a predictor of interpreter competence, suggesting that it may mitigate occupational pressure. However, emotional stability should not be understood as an innate disposition, but as the outcome of sustained emotional effort. Since emotions are not merely psychological states but also social and cultural practices that position individuals in relation to the world (Ahmed, 2004, p. 9), they are necessarily implicated in broader discussions of interpreting norms.

While Hochschild’s tripartite framework serves as the core analytical tool for this study, it is situated with other complementary theoretical perspectives. The Job Demands–Resources (JD-R) model (Bakker and Demerouti, 2007) conceptualizes emotional labor demands as occupational stressors that deplete psychological resources when insufficient buffering resources are available. In outsourced interpreting, the dual authority structure identified in this study may be understood as complex job demands while restricting interpreters’ resources. Conservation of Resources Theory (Hobfoll, 1989) similarly foregrounds the depletion of personal and social resources under sustained occupational pressure, offering an explanation of why emotional labor in inter-interpreting settings may function as a resource-replenishment strategy. Emotional Regulation Theory (Grandey, 2000) bridges Hochschild’s behavioral categories with cognitive and physiological self-regulation processes. This study focuses primarily on emotional management while invoking complementary frameworks where relevant to enrich the interpretation of findings.

Existing studies have examined disruptive stimuli that influence interpreting performance (Roziner and Shlesinger, 2010; Korpal, 2017), yet insufficient attention has been paid to the emotion regulation and emotional performances through which interpreters respond to such stimuli. Addressing this dimension offers a productive perspective for understanding the profession.

### 2.2 Emotional labor seen and unseen

Interpreters are emotionally affected by their work (Doherty et al., 2010; Hale and Napier, 2016), and they could adopt task-oriented coping and emotion-oriented coping to handle the adverse impact (Korpal, 2021). Current studies concentrate on specific settings such as medical and court interpreting, where power asymmetries are salient and emotional labor of interpreters can be read as internalized response (Baumgarten, 2017). In the context of court interpreting, Carstensen and Dahlberg (2017, pp. 53–58) find that interpreters could feel exposed or vulnerable while interpreting, and that they sometimes use distancing as a strategy to handle negative emotional experiences that seem to be a part of their work conditions. Recognizing interpreting demands conscious emotional strategies, Tekgül (2020, p. 7) identifies emotional mirroring and amplification as methods interpreters adopt to comply with professional roles and fulfil organizational goals in faith-related interpreting through ethnographic method. Based on an interview with freelance and staff interpreters, Ayan (2021) finds that interpreters have internalized managing their own feelings as a form of neutrality (2021, pp. 136–142). While existing research has explored the emotional labor interpreters undertake during interpreting, the unseen aspects of their professional lives, particularly the periods before and after interpreted encounters, remain underexplored.

Emotional labor is a lived experienced for interpreters not only during interpreting, but also in off-interpreting settings. The emotional labor exerted between interpreting encounters has only been mentioned in passing in existing research. While affirming that emotions are constantly present in interpreting, Kara and Nordberg (2023, pp. 572–73) touch upon the intense effort interpreters invest in processing emotions afterwards and the potential for dealing with these emotions collaboratively with other interpreters (c.f. Hoza, 2010). Reaffirming interpreters are not machine-like, emotionless beings during interpreting, Rosendo (2021, pp. 47) argues that interpreters could develop different attitudes that influence their perception and interpretation of reality based on their previous experiences. This suggests a possible connection between the emotional labor interpreters perform during interpreting and in off-interpreting settings.

The norms, client expectations, and interpreter behaviors in conference interpreting vary significantly depending on who employs the interpreters, for whom, and in what context (Ayan, 2021, pp. 128). Freelance interpreters, who typically work with a particular team only for the duration of a contract, often operate in solitude or small circles. Possibly harboring an elitist attitude, interpreters are at times troublesome, aggressive and do not fit in (Moser-Mercer, 2005, pp. 86). Regular conferences usually last from half a day to a few days, providing interpreters with few opportunities to engage with stakeholders. This combination of employment form and interpreter’s temperament allow limited possibility for the observation of their emotional state. In outsourced interpreting, interpreters must interact with grantors, agencies and fellow interpreters over an extended period, and behave in a way that befit their employment status as well as institutional expectations, necessitating emotional labor on different levels and occasions. The emotional labor in off-interpreting settings resulting from contractual disenfranchisement is often implied but not sufficiently discussed. Examining court interpreters who have to handle agencies and clients as well as poor organization of work, Korpal (2021, pp. 558) points out that some aspects of interpreting may be both cognitively demanding and emotionally taxing, potentially leading to overall dissatisfaction with the profession. Considering collaboration is present in interpreting before and after the communicative event (Jiménez-Crespo, 2015, pp. 76), interpreters have to avoid clashes and confrontations, necessitating at least some degree of feeling management.

To reveal the unseen emotional labor beyond the visible scope of interpreted encounters, this research examines the experience of 52 professional interpreters at the Hangzhou Asian Games 2023. In the context of outsourced interpreting services, interpreters have to follow institutional guidelines and submit to the management of the language company. This dual supervision structure imposes additional institutional pressures on interpreters, who subsequently experience greater emotional dissonance. Although interpreters maintain a de facto employment relationship with the language company, they strive to maintain positive relationships with all stakeholders, including the Organizing Committee, the language company, and fellow interpreters, to secure professional rewards such as positive evaluations, recognition, work allocation, and substantial earnings (Giustini, 2023, pp. 427). As a result, the negative effects of emotional dissonance do not immediately impact interpreters’ performance but instead lead to burnout, which is managed discreetly in post-interpreting settings (c.f. Zapf, 2002, pp. 237). This paper aims to unveil the unseen emotional labor outside interpreted encounters, and argues that such emotional labor mitigates the negative impact of outsourced interpreting on interpreter’s well-being and professional practice.

## 3 Research design

Following a poststructuralist approach, Bahadır (2005, pp. 816) conceptualizes interpreters as ethnographers and interpreting researchers as anthropologists, emphasizing the necessity to look at the ‘fieldwork’ of interpreters, ‘the actually lived experiences show us a path out of endless (semi) theoretical discussions in a prescriptive vacuum’. Ethnographies that extend beyond the level of interpreting interaction are scarce, yet they are crucial for gaining deeper insights into ways of speaking of interpreters and the communities in and for which they work (Angelelli, 2015, pp. 150). Recent years have witnessed a growth of ethnographies in interpreting research (Inghilleri, 2021; Giustini, 2019; Shen and Gao, 2025), echoing the call of Ayan (2021, pp. 143) for more analyses of interpreters’ first-hand experience.

Autoethnography, as a strand of ethnography, positions the researcher’s lived experience at the center of analysis in order to illuminate broader cultural and institutional processes (Ellis et al., 2011). The author collected data for four months from the preparatory phase to after the closing of the Asian Games. By participating as both a researcher and one of the 52 interpreters, the author adopts an autoethnographic approach to data interpretation. This dual role aligns with what Anderson (2006, pp. 390) refers to as ‘analytic autoethnography’, which allows a vantage point for accessing data and the provision of insider meanings of the sociocultural contexts in which we live (cf. Hokkanen, 2017). Such insider positionality inevitably carries epistemological implications. The researcher’s own emotional experiences as an interpreter informed both the thematic orientation of the study and the selection of illustrative cases. Consistent with the analytic autoethnographic tradition, this reflexive dimension is treated not as a source of bias but as an analytical resource.

To operationalize the analysis of autoethnographic data, a sequential explanatory mixed-methods design was adopted (Creswell and Plano Clark, 2018), in which quantitative sentiment analysis of interpreter communications is conducted first and is followed by qualitative semi-structured interviews designed to deepen and contextualize the numerical findings. This design is particularly suited to investigating emotional labor in outsourced interpreting, as such experiences are simultaneously quantifiable at the aggregate level and deeply personal in nature. The sentiment analysis identifies the emotional contours across the Games that warrant explanation, while the interviews provide the interpretive depth needed to understand the social and professional mechanisms behind these patterns (Tashakkori et al., 2021). Together, they ensure that neither statistical patterns nor individual accounts are interpreted in isolation, thereby strengthening both methodological rigor and analytic transparency.

Even though autoethnography does not refrain from the researchers’ positionality on assuming multiple roles, two reflexive procedures were implemented to manage the epistemological risks. First, the quantitative sentiment analysis phase was completed before interview questions were finalized, ensuring that the qualitative strand was designed to interrogate rather than simply confirm emerging patterns. Second, one interviewee was invited to review a thematic summary of their interview data as a form of member-checking.

### 3.1 Sentiment analysis

A sentiment analysis is performed on the interpreters’ working group chat on WeChat, China’s most popular instant messaging app, from July to October. A sentiment analysis software, Weiciyun, is utilized to process the group chat. Weiciyun is a Chinese-language text analytics platform that performs sentiment classification through a lexicon-based approach, assigning polarity scores to tokens drawn from a curated dictionary of affective terms calibrated for contemporary Chinese digital communication. Lexicon-based sentiment analysis has been widely applied to Chinese social media and text data and constitutes an established methodological option when the analyst requires scalable, automated affective coding of large corpora (Liu, 2012). The tool’s suitability for the present data rests on its coverage of vernacular and emoji-equivalent expressions that carry pragmatic affective meaning that standard text would not capture.

A total of 82,209 Chinese characters over four months were logged. After data cleansing to remove duplicates, unrelated comments, and system-generated messages, 39,477 characters (21,980 words) were analyzed. The group chat is segmented into four phases for a global content and sentiment analysis: pre-Games, Games time, the last day of the Games, and post-Games. The results are presented in Table 1.

**Table 1:** Descriptive statistics on sentiment.

Period	Word count	Positive words (%)	Neutral words (%)	Negative words (%)	Sentiment score
Pre-games	3,194	50.82	31.23	17.95	3.87
Games time	12,255	36.30	40.23	23.47	2.48
Last day	2,015	65.39	25.96	8.65	7.90
Post games	3,796	51.44	30.67	17.89	3.85
Total/average	21,980	42.73	36.02	21.25	6.74

**Figure 1:**
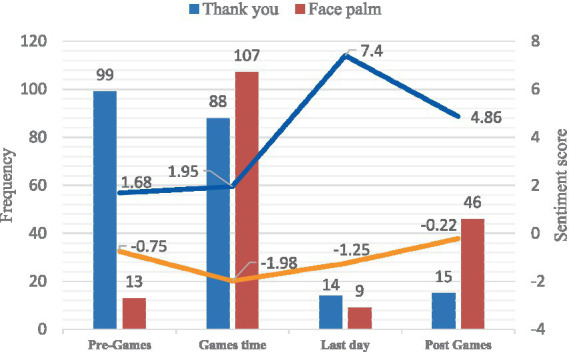
Word frequency and sentiment score of thematic words.

Frequency and TF-IDF analysis identify two dominant emotion-signifying words *谢谢* ‘thank you’ (*n* = 216, tf-idf = 0.02994) and *捂脸*^1^ ‘face palm’ (n = 175, tf-idf = 0.02981). ‘Thank you’ generally indicates positive emotion, while ‘face palm’ is an emoticon used to soften criticism or negative comments, thus a symbolizing negative emotion. A sentiment analysis over the two themes were then conducted. The frequency and sentiment score of the two thematic words for the four phases are reflected in Figure 1.

It is important to note that in Chinese workplace digital communication, both markers carry pragmatic flexibility. ‘Thank you’ may reflect genuine appreciation, social compliance, or deliberate self-presentation, while ‘face palm’ may function as ironic softening. These two markers are therefore analyzed as behavioral signals consistent with certain emotional orientations rather than as unambiguous indicators of subjective emotional states. The broader macro-level sentiment analysis (covering the full 21,980-word corpus) provides the overarching affective landscape, within which these markers serve as illustrative anchors. Conclusions derived from these markers are presented as patterns consistent with emotional labor rather than as direct evidence of the psychological processes underlying it.

### 3.2 Interview

Acknowledging the limitations of lexicon-based approaches, which may misinterpret highly context-dependent utterances due to inadequate coverage of affective language unique to professional interpreting contexts, the qualitative component of the mixed-method design is adopted as a triangulation strategy to support and contextualize the classification results. Semi-structured interviews were conducted with two interpreters selected on the basis of the author’s observations as representative of contrasting positive and negative emotional experiences. This purposive sampling strategy follows the logic of maximum variation sampling (Patton, 2002), whereby cases are selected not for statistical representativeness but for their theoretical capacity to illuminate the phenomenon under investigation across its most divergent manifestations. In interview-based qualitative research, sample adequacy is evaluated not by size per se but by the analytical significance of the selected cases (Robinson, 2014). The two interpreters are therefore treated as critical cases whose experiences collectively capture the range of emotional labor outcomes observable within the broader cohort of 52 interpreters.

The designation of these two interpreters as critical cases rests on two grounds. First, as a researcher-participant embedded in the cohort for four months, the first author had sustained direct contact with all 52 interpreters in a shared workspace, enabling continuous observation of their emotional trajectories. Second, the emotional experiences of the broader cohort were assessed collectively through the sentiment analysis of the shared group chat, a medium in which all 52 interpreters participated, providing an aggregate emotional baseline against which individual accounts could be situated. The interviews are not intended to represent the diversity of the full cohort statistically but to illuminate the mechanisms behind the emotional patterns identified at the aggregate level. This dyadic contrastive design enables an in-depth examination of the mechanisms underlying both adverse and enriching emotional trajectories. The findings are not intended for statistical generalization but to generate theoretically transferable insights into the emotional dynamics of outsourced interpreting in large-scale sporting events.

Interpreter Jake (pseudonym) is a freelance professional interpreter with 5 years of experience who received a negative evaluation from the Organizing Committee. As a result, Jake (pseudonym) was benched for the rest of the Games and served as a backup for other interpreters. This proved to be a traumatic experience for Jake (pseudonym) and incurred a series of emotional efforts for its reconciliation. Interpreter Dan (pseudonym) is a novice interpreter who was still in a master program during the Games time, but was convoked for being certified at CATTI-1 for interpreting. Positive feedbacks from the Organizing Committee and the interaction with professional interpreters constituted as a positive emotive experience which further prompted Dan (pseudonym) decision to pursue another degree in Conference Interpreting in a world-renowned interpreting institution.

The interview questions, informed by theory and literature review, were developed from the quantitative analysis results and divided into three sections: understanding power asymmetries in outsourced interpreting and emotional states during and after interpreting. One interview was arranged at a private office and the other online. Both interviews were audio-recorded, transcribed, and conducted in Chinese, with translations into English. The interpreters’ responses provide supporting evidence for the understanding of the quantitative sentiment analysis data.

## 4 Analysis and discussion

To better understand the emotional effort of interpreters, we propose a classification of ‘intra-interpreting’ and ‘inter-interpreting’ settings. Settings as contextual categories specific to the practice of interpreting. Intra-interpreting settings refer to the periods of active interpreting assignments; inter-interpreting settings denote the intervals when interpreters are in between assignments within the validity of contract. They provide a contextual mapping for the inquiry of interpreters’ emotional labor.

In the context of the Asian Games, the language company assumes the direct management of interpreters, conveying directives and evaluations from the Organizing Committee. Under such power structure, the language company is the tangible superior for interpreters, while the Organizing Committee represents a conceptual higher authority. Drawing from sentiment analysis conducted on the group chat, this section investigates interpreters’ emotional efforts in response to the power dynamics in outsourced interpreting.

### 4.1 Self-presentation on purpose

Emotional labor is the emotional effort paid during interactions with customers or clients, but it could also entail one’s interaction with employers (c.f. Tschan et al., 2005). Such interaction, however, often remains hidden as they usually occur backstage of interpreting events. In the Asian Games, interpreter’s emotional labor primarily targets the language company, which acts as the intermediary between the Organizing Committee and interpreters, thereby avoiding displeasing the entity responsible for interpreting assignments.

The language company set up the WeChat group at the end of July, approximately two months before the Games. Communications during this period included the dissemination of preparatory materials, term quizzes, and administrative announcements. Given the absence of in-person contact between interpreters and employers/clients, interpreters’ sentiments during this phase can be taken as a benchmark for their emotional fluctuations in subsequent periods. Sentiments during the pre-Games phase portray interpreters as proactive and well-adjusted professionals. Positive words account for 50.82%, followed by 31.23% of neutral words and 17.95% of negative words. The most frequent words expressed during this stage is ‘thank you’ (*n* = 99), which often comes on the heels of *收到* ‘copy that.’ Such response is commonly observed when project managers issue notices or distribute glossaries. The reasons behind the overall positive expression can be attributed to the lack of in-person interaction, common social courtesy, and deliberate self-presentation. While the first two reasons are dictated by social context, the latter is determined by interpreters.

Speaking up in workplace group chat implies drawing attention to oneself. Wei et al. (2015, p. 1) point out that supervisory delegation allows employees to perceive their positive voice with high efficacy and low risk, even in environments characterized by power distance. In outsourced interpreting, the language company assumes the delegated supervisory role from the Organizing Committee. Therefore, interpreters are more likely to speak up when their voice could enhance their visibility while at the same time risk-free. It appears gratuitous for approximately 20 interpreters to inundate the group chat with identical responses of ‘copy that, thank you’ despite explicit instructions indicating ‘no reply needed.’ Possible reasons to explain such behavior is that professional etiquette, collectivist communication norms, and deliberate impression management may operate simultaneously and are not mutually exclusive.

However, a key feature of emotional labor is that it is manufactured and staged while the aim is to appear authentic (Koskinen, 2020, pp. 30). A possible explanation for their display of authenticity in this case is the power dynamics in outsourced interpreting. In the conventional interpreting market, interpreters are accustomed to *ad hoc* freelance work that usually only involves contact with the client. In outsourced interpreting, the two-party power relationship now transforms into a three-party power structure, in which interpreters are no longer freelance but quasi-employees that report to language companies as their superiors. Under such circumstances, interpreters are motivated to appear authentic, primarily through minimal cost online verbal participation via surface acting. While this form of emotional labor may not yield immediate tangible reward, it serves as a means for interpreters to accumulate social capital for future engagements through the commercialization of emotions. Interpreter Jake (pseudonym) says: “.

“I know the ultimate client is the Organizing Committee, but my direct report is the language company. I don’t think we are on equal footing here as I’m the party B. I can always be replaced by other interpreters but I can’t find another language company to work on the Games. When you are a Party B, you must find ways to secure the job from Party A.”

Interpreter Dan (pseudonym) says:

“I’m not quite clear on the relationship between them, all I know is that I answer to my project manager, who’s from the language company. I am on a lower position in relation to the language company as they are the one who assign jobs and evaluate performances. I’m their laborer.”

Interpreter Jake (pseudonym) highlights the asymmetrical power dynamics, recognizing the language company as the direct employer with the authority to determine job assignments, emphasizing the need to secure employment within this hierarchical structure. Interpreter Y, while lacking clarity regarding the relationship between the Organizing Committee and the language company, acknowledges their subordinate position to the language company, which dictates job assignments and evaluates performance. Both interpreters are aware of the language company’s decisive role in their professional activities during the contract period.

In outsourced interpreting, power dynamics shift downward to the language company, making the latter the de facto employer that handles contract and remuneration. Under such circumstances, interpreters endeavor to avoid being perceived as aloof or difficult to work with. Through making a good showing of ourselves, we can have a good face when we fit an image others have of our profession (Goffman, 1967, p. 5). The display of socially desirable attributes entails emotional labor from the interpreters. While surface acting remains relatively straightforward at this stage, maintaining a congenial demeanor during the Games becomes increasingly challenging as various factors contribute to emotional dissonance.

### 4.2 Discontent in disguise

Interpreters exhibit the lowest level of satisfaction during the Games period. They were most active during the 14-day event, generating 12,255 words in the group chat. However, the proportion of positive words dropped to 36.3%, the lowest across all periods, from 50.82% in pre-Games time. Meanwhile, neutral and negative words account for 40.23 and 23.47%, respectively, the highest percentages observed. The overall sentiment score is 2.48, also the lowest, although still positive. The 1.39-point decline from the baseline score of 3.87 in the benchmark period of pre-Games time can be attributed to both intra-interpreting and inter-interpreting factors.

The Organizing Committee mandates that interpreters maintain neutrality regardless of the emotion of speakers during intra-interpreting settings. Instead of trying to embody speaker’s emotions or stroke their egos, interpreters now have to inhibit or disassociate them. In managing their emotions during interpreting, interpreters have to employ surface acting, assuming a neutral and indifferent voice to conform to institutional requirements. While such requirement can be understood as a prevention technique for interpreters to not distort the message (Zwischenberger, 2022, pp. 232), it is also a form of risk-prevention method for interpreters to distance themselves from emotional upheavals and thus avoid emotional overload (Koskinen, 2020, pp. 104).

In the case of interpreting for sporting events, stripping away emotions can be a self-defeating practice. Interpreter Dan (pseudonym) says: “*I try to be indifferent most of the time, but when they get emotional, I get emotional. After all, how do you not feel overwhelmed when an athlete says with tears that she’s been training for this medal for 10 years*?” The conflict between institutional demands and human nature forces interpreters to regulate their emotions consciously. The idea of putting on the mask of professionalism to prevent emotional overload in fact, can paradoxically impose additional emotional effort. In rare cases when interpreters are faced with criticism or confrontation in intra-interpreting settings, they would often employ shock-absorbing strategy, prioritizing job completion at the expense of their emotional well-being. These emotional labor in intra-interpreting settings are managed by interpreters through shutting off emotions, deferring emotions, and processing them later (c.f. Moesby-Jensen and Nielsen, 2015).

Emotional labor in intra-interpreting settings could lead to a subsequent round of emotional labor in inter-interpreting settings. To deal with the inhibited emotions felt in intra-interpreting settings, interpreters frequently articulate genuine feelings in inter-interpreting settings with fellow interpreters. The feelings interpreters mostly expressed include frustration, dissatisfaction and grievance. Korpal and Mellinger (2022, pp. 275) point out that emotionally-charged interpreting can be stressful for interpreters, who may resort to self-care strategies such as awareness, social support and spirituality to mitigate stress. Both the Organizing Committee and the language company are indifferent to interpreters’ emotional state as long as the interpreting is successful. Interpreters, however, did not receive any prevention or coping strategies on emotional dissonance, and they lack a support system due to professional isolation (Liu, 2024, pp. 77). All interpreters interviewed admit that they would recount unjust or challenging incidents to other interpreters, seeking resonance, empathy, or a sense of belonging. The expression of genuine emotions as a form of emotional labor can have a mitigating effect on emotional dissonance as it fosters a sense of mutual support. It should also be noted that they admit they only share genuine feelings with interpreters they trust to avoid being disempowered by other ill-intentioned interpreters who want to showcase individual professionalism to the language company by tattling the complaint of their colleagues (c.f. Giustini, 2023).

The power structure of outsourced interpreting could be another cause of emotional dissonance, which often manifest in inter-interpreting settings. Interpreters are subjected to a multi-faceted evaluation mechanism, with quality managers, Organizing Committee staff, and direct users of interpreting services all providing feedback. Criticism from the Organizing Committee is detrimental to interpreters, but negative feedback in general could hurt interpreters’ prospects in securing more opportunities during the Games. Interpreters may be benched, and in some cases, be prevented from servicing the ensuing Para Asian Games. Interpreters are under constant stress as they do not always know if their performance is under evaluation, especially when the evaluators may just be a bilingual speaker rather than a professional(Shen, 2025; Shen and Gao, 2023). Such mechanism could exact an emotional toll on interpreters regardless of their performance. Interpreter Jake (pseudonym) says:

“This was my only negative review in 2023. It was an important press conference and many were there to monitor. It was very traumatic for my ego when I learned that I was dismissed for future events. It took me a long time to recover from this frustration, but I felt better when I found out I was not the only one criticized and some interpreters who are not as good as me only got a safe pass because they were not evaluated at their turn.”

The emotional toll the evaluation mechanism had on interpreters is not only limited to those who were negatively evaluated. Even acclaimed interpreters find the evaluation system stressful. Interpreter Y remarks:

“I understand why there is an evaluation system. They sign our pay checks, of course they are entitled to evaluate the services they get. I have no problem with being evaluated by big-shot interpreters, but I don’t feel well when evaluated by amateurs.”

In addition to evaluations, interpreters are also subjected to quasi-full-time employment norms. They are asked to punch the clock via a mobile app within the perimeters of interpreting venues, adhere to code of conduct for contractors, and work in unhealthy hours as some events conclude at midnight. They lack negotiating power over labor conditions, as the language company itself is subject to the Organizing Committee’s arrangements. The discontent interpreters experienced is supposed to be reflected by negative emotions. However, the sentiment analysis of group chats reveals that positive sentiment persists, albeit at the lowest levels. This discrepancy suggests that at least some form of emotional labor has been taking place, either by the suppression of genuine feelings online or their display with peers.

Speaking up in group chat is a decision that requires at least some degree of contemplation. During the Games time, interpreters continue to express a lot of ‘thank you’ (*n* = 88). However, the number of ‘face palm’ (*n* = 107) reached a record high with a sentiment score registering −1.98, the lowest at all times. Employees are often reluctant to speak up in group chats due to consequences such as fear of demotion, job loss, being perceived as offensive and disrespectful, or would not make a difference (Tarim, 2017, pp. 14). As ‘face palm’ has a mitigating effect over criticism or a negative comment, it has the potential to be seen as a form of conscious emotional labor by interpreters as they value on superficial harmony and ambient group voice climate. The data support the emotional labor interpretation as plausible and consistent, but do not preclude the alternative that such behavior reflects a learned communicative convention. In practice, ‘face palm’ is generally attached to interpreter’s personal appeals, potentially offensive opinions, or assistance requests. Giustini (2019, pp. 186) point out that interpreters are capable of handling emotions with reason, use professional skills to re-balance power relations, positively impact users and ensure successful communication during interpreting. This study finds that emotional management in intra-interpreting settings are transferrable and also utilized in inter-interpreting settings to mediate their relationship with other stakeholders. Specifically speaking, surface acting is the most-observed emotional management technique to mask negative emotions during Games time when engaging with the language company.

### 4.3 Enthusiasm on display

In addition to conscious emotional labor, interpreters could also engage in unconscious emotional labor in outsourced interpreting. The sentiment score of the last day of the Games is at an all-time high of 7.9. Positive words account for 65.39% of the total, and only 8.65% are negative words. The stark contrast between sentiment on the last day and the pre-Games (*n* = 3.87) and post-Games (*n* = 3.85) periods warrants careful interpretation. The observed pattern may be explained by deep acting, self-induced emotional expressions rooted in professional inculcation and role expectations. It is equally plausible that expressions of appreciation were more genuinely felt as a natural emotional response to the conclusion of a demanding professional experience. Deep acting, as a psychological process of emotional internalization, cannot be directly inferred from group chat behavior alone. The data provide behavioral correlates rather than direct evidence of emotional internalization. Authentic positive affect, socially compelled performativity, and deep acting may all be operating simultaneously, and the analysis is presented on that understanding.

During the post-Games period, interpreters no longer had contractual obligation with the language company. Indicators during this phase closely mirrored those of the benchmark period of pre-Games, with overall sentiment at 3.85 and positive, neutral, and negative words at 51.44, 30.67, and 17.89%, respectively. Notably, the use of ‘face palm’ emoticons increased from 13 in the pre-Games period to 46 in the post-Games period. This likely reflects interpreters’ greater ease in expressing emotions, now free from the power asymmetry. Interpreters still employed face-saving strategies, but their expressions of ‘thank you’ are more genuine, with a sentiment score of 4.86, compared to 1.95 during the Games and 1.68 pre-Games. However, on the last day, the sentiment score for ‘thank you’ peaked at 7.4, thus worth closer examination.

On the last day, most interpreters had no job assignments and were preparing to leave Hangzhou after clocking out. The day was marked by repeated expressions of gratitude and appreciation. Employees are likely to speak up in group chat when they feel comfortable, and if they perceive high efficacy and low risk associated with such behavior (Premeaux, 2001; Wei et al., 2015). Beyond genuine appreciation, two factors likely drove the high level of emotive expression. First, interpreters are under heavy peer pressure to express gratitude in the group chat, as not doing so risk them looking aloof and distant. Second, the termination of interpreting contract with the language company reduced concerns about repercussions from speaking up. Besides, there would not be any negative consequences when expressing appreciation for colleagues and the language company. Thus, it is almost a wasted opportunity should one not speak up in this occasion.

Interpreters’ surface enthusiasm could be a result of acculturation after years of professional practice. The interpreting market has inculcated them into presenting a socially desirable self to attract, please and appease clients. Interpreters are acculturated to a professional conduct of ‘inconspicuous display of individual excellence’ in their labor to attract clients’ attention following the rationale to secure professional reward (Giustini, 2023, pp. 436). The individual excellence includes not just interpreting competence, but also interpersonal skills that make up interpreters’ professional skills. Stimulated by emotional labor, individuals classify themselves into an imagined, ascribed collective stereotype to inculcate a sense of belonging, which allows them to feel most authentic when they are conforming to role expectations (Ashforth and Humphrey, 1993, pp. 98). Combined with what may be genuine nostalgia for the Games, interpreters appear to have amplified their expressions of appreciation in ways consistent with the deep acting process, aligning emotive expression with their professional role identity.

## 5 Implications and recommendations

### 5.1 Implications

Theoretically, this study introduces the distinction between intra-interpreting and inter-interpreting settings, thereby extending the framework of emotional labor beyond immediate service encounters to the broader relational and contractual spaces surrounding them. Emotional labor in professional linguistic services is therefore not confined to the interpreting event itself, but unfolds across the entire temporal trajectory of an outsourced contract. This conceptual extension may also be applicable to other professional service sectors characterized by outsourced employment arrangements. By empirically demonstrating the relationship between power asymmetry in outsourced employment and intensified emotional labor demands, the study identifies a dual-authority structure in which interpreters are simultaneously accountable to language companies and, indirectly, to organizing committees. This arrangement generates a compounded form of emotional pressure that has received limited attention in interpreting studies. In doing so, the study echoes Zapf’s (2002) argument that organizational power differentials structurally shape emotion work demands, while extending this framework to a non-Western, event-based outsourcing context.

Practically, the study offers managerial insights for language companies and service commissioners. As the direct managers of interpreters in outsourced arrangements, language companies may function simultaneously as sources of emotional pressure and providers of emotional support. The findings suggest that language companies may benefit from implementing pre-assignment briefings that explicitly acknowledge the emotional demands of large-scale event interpreting and establishing peer communication channels that are clearly demarcated as safe spaces for genuine emotional expression. Proactive management of interpreter wellbeing may yield downstream benefits for service quality. Service commissioners may wish to consider requiring that language service contracts include provisions for interpreter wellbeing and establish feedback mechanisms through which interpreters can raise concerns without professional repercussion.

### 5.2 Recommendations

Based on the findings, recommendations are made in terms of interpreter’s evaluation, communication and training. First, opaque and *ad hoc* evaluation are a particular source of emotional distress and undermine interpreters’ professional confidence without serving legitimate quality assurance goals. Standardizing evaluation criteria and communicating them transparently to interpreters may help address a central source of emotional strain. Second, dedicated communication spaces independent of employer-monitored channels are recommended to enable interpreters to share genuine emotional experiences with colleagues without professional risk. The present study illustrates that informal peer communication already fulfills this function, thus formalizing and protecting such spaces may enhance its effectiveness, though this was not directly evaluated in the study. Third, training programs could address the emotional demands of outsourced interpreting, including strategies for managing emotional dissonance, recognizing the signs of emotional overload, and accessing support resources. Such preparation may enable interpreters to engage in more sustainable and deliberate forms of emotional self-regulation rather than relying primarily on improvised coping strategies.

## 6 Conclusion

This research unveils the unseen emotional labor in outsourced interpreting by examining the experiences of 52 Chinese-English interpreters during the 19th Hangzhou Asian Games. Using an autoethnographic lens, it analyses the sentiment of interpreters in the group chat through sentiment analysis and follow-up interviews. Interpreters are subjected to evaluations from both the Organizing Committee and the language company who manages them. Recognizing interpreters have to deal with a two-fold power asymmetry, this research classifies intra-interpreting and inter-interpreting settings to understand the emotional labor of interpreters in response to such power structure. These are contextual categories that map the temporal ecology of interpreting work rather than new theoretical constructs of emotional labor. In managing their emotions in intra-interpreting settings, interpreters employ surface acting, assuming a neutral and indifferent voice to meet institutional requirements. The study finds that the emotional labor during interpreting may lead to a subsequent round of emotional labor in inter-interpreting settings, along with other factors in outsourced interpreting that contribute to emotional dissonance, such as dual evaluation mechanism and less-than-ideal work condition. While outsourced interpreting guarantees institutional well-being at the cost of interpreter’s individual well-being and job satisfaction, interpreters engage in invisible emotional labor that appears oriented toward mitigating emotional dissonance, as reflected in behavioral patterns consistent with coping, out of inculcation and professional reward. The emotional labor afforded is not only a response to the superficial demands of the interpreting task but also an attempt to address the negative impact of outsourced interpreting on their well-being and job satisfaction.

Limitations of the present study should also be acknowledged. First, the study is based on a single large-scale event within a specific national and professional context, thus limits its universality. Second, sentiment analysis provides only an indirect and aggregated measure of emotional expression rather than a direct measure of internal emotional states. Third, although the follow-up interview data are valuable for contextualizing sentiment patterns, the interview sample remains small. A larger and more systematically selected interview sample would strengthen the robustness of the interpretations. Given the unique employment structure of the Asian Games, further research could explore emotional labor in broader outsourced interpreting settings to generalize these findings. This study may inspire further investigation of interpreters’ emotional labor across diverse employment structures and interpreting phases.

## Data Availability

The original contributions presented in the study are publicly available. This data can be found here: https://cstr.cn/31253.11.sciencedb.41043, https://doi.org/10.57760/sciencedb.41043.

## Footnotes

1 Face palm is an emoticon that is system-designed to be triggered by directives ‘/face palm’. The transcription of chats would return the emoticon to word directives, thus allowing emoticons to be analysed as words
